# Beneficial effects of melatonin on liver fibrosis: A systematic review of current biological evidence

**DOI:** 10.1002/jcp.30735

**Published:** 2022-04-11

**Authors:** Beatriz San‐Miguel, Paula Fernández‐Palanca, José L. Mauriz, María J. Tuñón, Javier González‐Gallego

**Affiliations:** ^1^ Institute of Biomedicine, (IBIOMED) University of León León Spain; ^2^ Centro de Investigación Biomédica en Red de Enfermedades Hepáticas y Digestivas (CIBERehd) Instituto de Salud Carlos III Madrid Spain

**Keywords:** antioxidant, cirrhosis, hepatic fibrosis, liver fibrosis, melatonin

## Abstract

Hepatic fibrosis is a reversible response to either acute or chronic cellular injury from a wide variety of etiologies, characterized by excessive deposition of extracellular matrix resulting in liver dysfunction and cirrhosis. Melatonin (*N*‐acetyl‐5‐methoxytryptamine), the main product secreted by the pineal gland, is a multitasking indolamine with important physiological functions such as anti‐inflammatory and antioxidant actions, modulation of circadian rhythms, and immune system enhancement. Among the numerous biological activities of melatonin, its antifibrotic effects have received increasingly more attention. In this study, we performed a systematic review of publications of the last 10 years evaluating the mechanisms of action of melatonin against liver fibrosis. The study protocol was registered at PROSPERO (CRD42022304744). Literature research was performed employing PubMed, Scopus, and Web of Science (WOS) databases, and after screening, 29 articles were included. Results from the selected studies provided denoted the useful actions of melatonin on the development, progression, and evolution of liver fibrosis. Melatonin antifibrotic effects in the liver involved the reduction of profibrogenic markers and modulation of several cellular processes and molecular pathways, mainly acting as an antioxidant and anti‐inflammatory agent. In addition, the indolamine influenced different molecular processes, such as hepatocyte apoptosis, modulation of autophagy and mitophagy, restoration of circadian rhythms, and modulation of microRNAs, among others. Although some limitations have been found regarding variability in the study design, the findings here summarized display the potential role of melatonin in ameliorating the development of liver fibrosis and its possible progression to liver cirrhosis and hepatocarcinoma.

## 1 INTRODUCTION

The liver is a crucial organ that exerts detoxication actions of a great variety of molecules, such as protection from the harmful reactive oxygen species (ROS) generated during oxidative stress (Reyes‐Gordillo et al., 2017; Roehlen et al., 2020). Oxidative stress is a key factor in liver damage caused by a wide diversity of agents and contributes to virtually all conditions that compromise liver function, including ischemia‐reperfusion, nonalcoholic steatohepatitis, nonalcoholic fatty liver disease (NAFLD), liver fibrosis, liver cirrhosis and hepatocarcinoma (HCC) (Reyes‐Gordillo et al., 2017; Roehlen et al., 2020). However, not only oxidative stress contributes to liver fibrosis development. Hepatic stellate cells (HSCs) activation during liver injury is one of the main processes associated with the progression of hepatic fibrosis and has been established as an interesting target for antifibrotic therapies (Lee et al., 2015). Moreover, activated HSCs are responsible for producing extracellular matrix (ECM) components, whose progressive accumulation abolishes the liver architecture on physiological conditions, leading to liver fibrosis establishment (Lee et al., 2015; Roehlen et al., 2020).

During the fibrogenic process, inflammatory cells are recruited and promote the differentiation of HSCs into myofibroblasts responsible for collagen production (Roehlen et al., 2020). In case this profibrogenic response is not abrogated by tissue‐repair mechanisms, uncontrolled HSCs activation and collagen deposition can lead to loss of liver functionality and an uninhibited‐fibrosis (Lee et al., 2015; Roehlen et al., 2020). Subsequently, the evolution of liver fibrosis can trigger acute or chronic liver failure, cirrhosis, portal hypertension, and even HCC, being associated with a high probability of multiorgan failure and high mortality. Nonetheless, although there is increasing scientific evidence that suggests that liver fibrosis is a dynamic lesion and may become reversible, the only clear treatment to effectively prevent fibrosis‐associated mortality is the elimination of the causative agent (Lee et al., 2015; Roehlen et al., 2020).

Melatonin, also known as *N*‐acetyl‐5‐methoxytryptamine, is a product mainly secreted by the pineal gland and widely distributed in the body, being present in the bone marrow, skin, gastrointestinal tract, and liver, among other organs. The liver is the organ that accumulates the highest concentrations of melatonin, and the only one that metabolizes circulating melatonin (Mortezaee & Khanlarkhani, 2018). In addition to modulating several molecular pathways of inflammation, oxidative stress, apoptosis, and cell damage (Carbajo‐Pescador et al., 2013; Hu et al., 2016; Zhang et al., 2017), melatonin is also able to regulate circadian rhythms, and different studies have demonstrated the beneficial effects of this indolamine regarding fibrosis in different organs, including the liver (González‐Fernández et al., 2018; Zhang et al., 2017).

The protective role of melatonin in liver fibrosis seems to involve different cellular and molecular processes, including hepatocyte apoptosis, cholangiocyte proliferation, inflammation or activation of myofibroblasts, inducing ECM deposits and significantly reducing histopathological changes in liver tissue (Hu et al., 2016; Mortezaee & Khanlarkhani, 2018). Several studies suggest that melatonin could play a promising role in the treatment of liver fibrosis and other liver pathologies. Actually, beneficial effects of melatonin have been observed against hepatotoxicity (Yang et al., 2021), fulminant hepatic failure (FHF) (Crespo et al., 2016), NAFLD (Joshi et al., 2021), and HCC (Sánchez et al., 2018). Considering the broad spectrum of processes that melatonin is able to modulate, an increasing number of studies have focused recently on the study of melatonin effects in liver fibrosis (Mortezaee & Khanlarkhani, 2018).

In the present article, we systematically review the scientific literature published in the last 10 years that focuses on the main molecular and cellular mechanisms associated with the antifibrotic effects of melatonin and the potential use of this molecule to improve the treatment strategies in this hepatic pathology.

## 2 MATERIALS AND METHODS

### 2.1 Research strategy

This systematic review has been conducted following the Preferred Reporting Items for Systematic Reviews and Meta‐Analyses (PRISMA) guidelines (Page et al., 2021) (Tables S1 and S2). Additionally, the study protocol has been registered in the International Prospective Register of Systematic Reviews (PROSPERO), associated with the registration code CRD42022304744. To carry out an exhaustive search, the PubMed, Scopus, and Web of Science (WOS) databases were used, from the beginning until December 31, 2021, identifying a total of 153 articles, 65 from PubMed, 32 from Scopus, and 56 from WOS.

The search strategy followed was the following for each database:

1. PubMed: (“melatonin” [All Fields]) AND (“liver fibrosis” [All Fields] OR “hepatic fibrosis [All Fields]).
2. Scopus: TITLE‐ABS‐Key (“melatonin”) AND (“liver fibrosis” OR “hepatic fibrosis”).
3. WOS: TS = (“melatonin”) AND (“liver fibrosis” OR “hepatic fibrosis”).

### 2.2 Inclusion and exclusion criteria

We applied the following inclusion criteria to select the items: (1) use of liver fibrosis as a study model; (2) use of melatonin as a treatment either alone or in combination with other compounds; (3) use of in vivo and/or in vitro models. Articles that met the following criteria were excluded: (1) review or compilation articles; (2) congress or conference communications; (3) articles whose text was in a language other than English; (4) papers published before 2011.

### 2.3 Study selection

Study selection was performed independently by two authors; however, any disagreement was resolved by a third author.

Once the original articles were fully searched, duplicates were eliminated between databases, and the articles were screened. Subsequently, articles were selected based on the inclusion criteria and were individually evaluated; articles that met the exclusion criteria were discarded. Finally, all those articles that met the eligibility criteria were identified and included in the qualitative analysis.

### 2.4 Data extraction

The data of the articles included in the study were extracted by two independent authors, including the following aspects: name of the first author and year of publication, inducer of liver damage, experimental model (in vivo, in vitro), melatonin administration strategy, as well as modifications in the studied processes.

## 3 RESULTS

### 3.1 Study selection and characteristics

**Figure 1:**
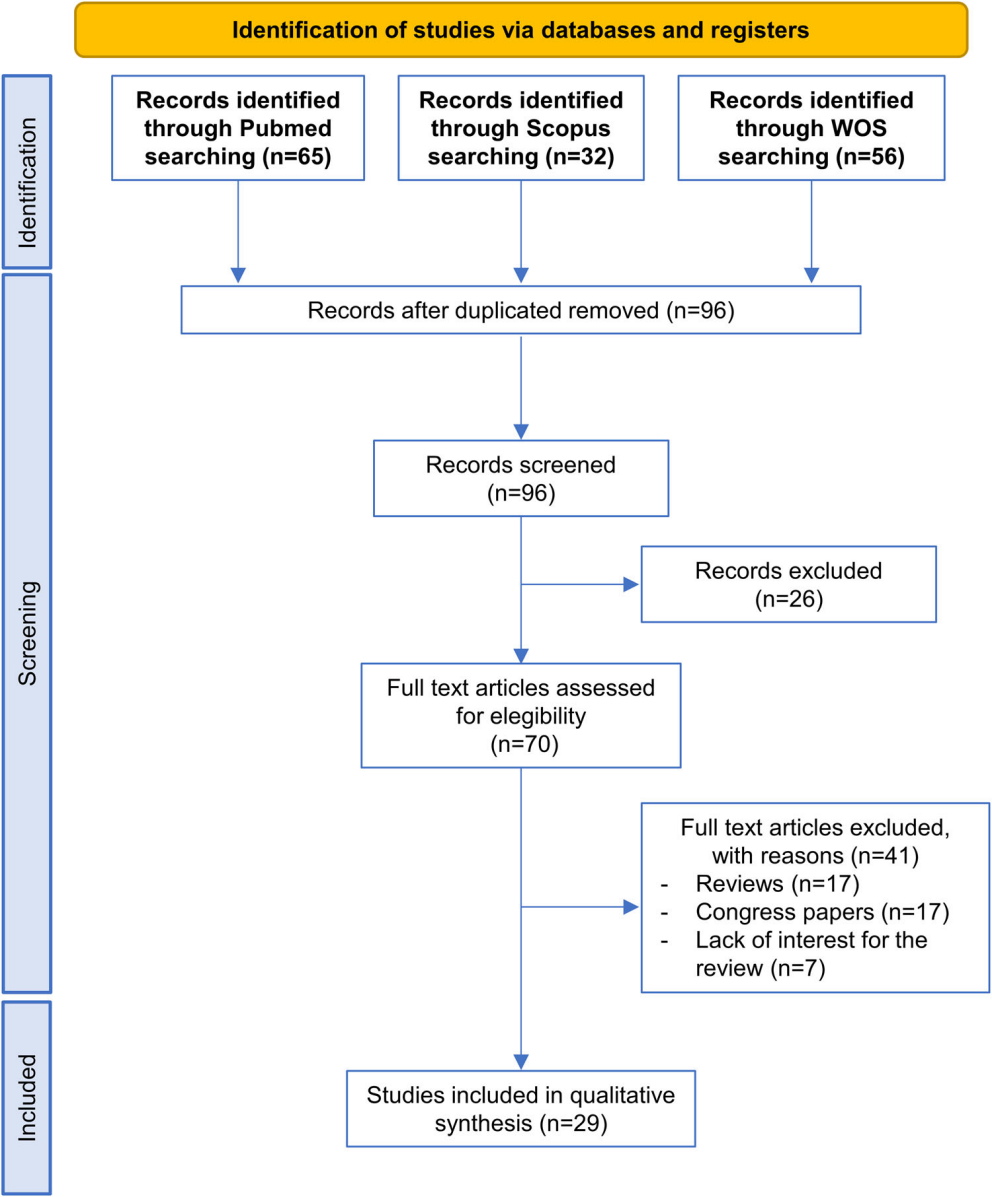
Flowchart of the study search conducted following the PRISMA guidelines. PRISMA, Preferred Reporting Items for Systematic Reviews and Meta‐Analyses; WOS, Web Of Science.

The complete process of study selection is defined in Figure 1. Briefly, the literature search conducted led to a total of 153 studies, being 96 after duplicates removal. Considering inclusion and exclusion criteria, 70 articles were fully screened, removing 41 that met exclusion criteria. After the complete screening, 29 original articles were identified as relevant studies that met the established goal and were included in this systematic review.

**Figure 2:**
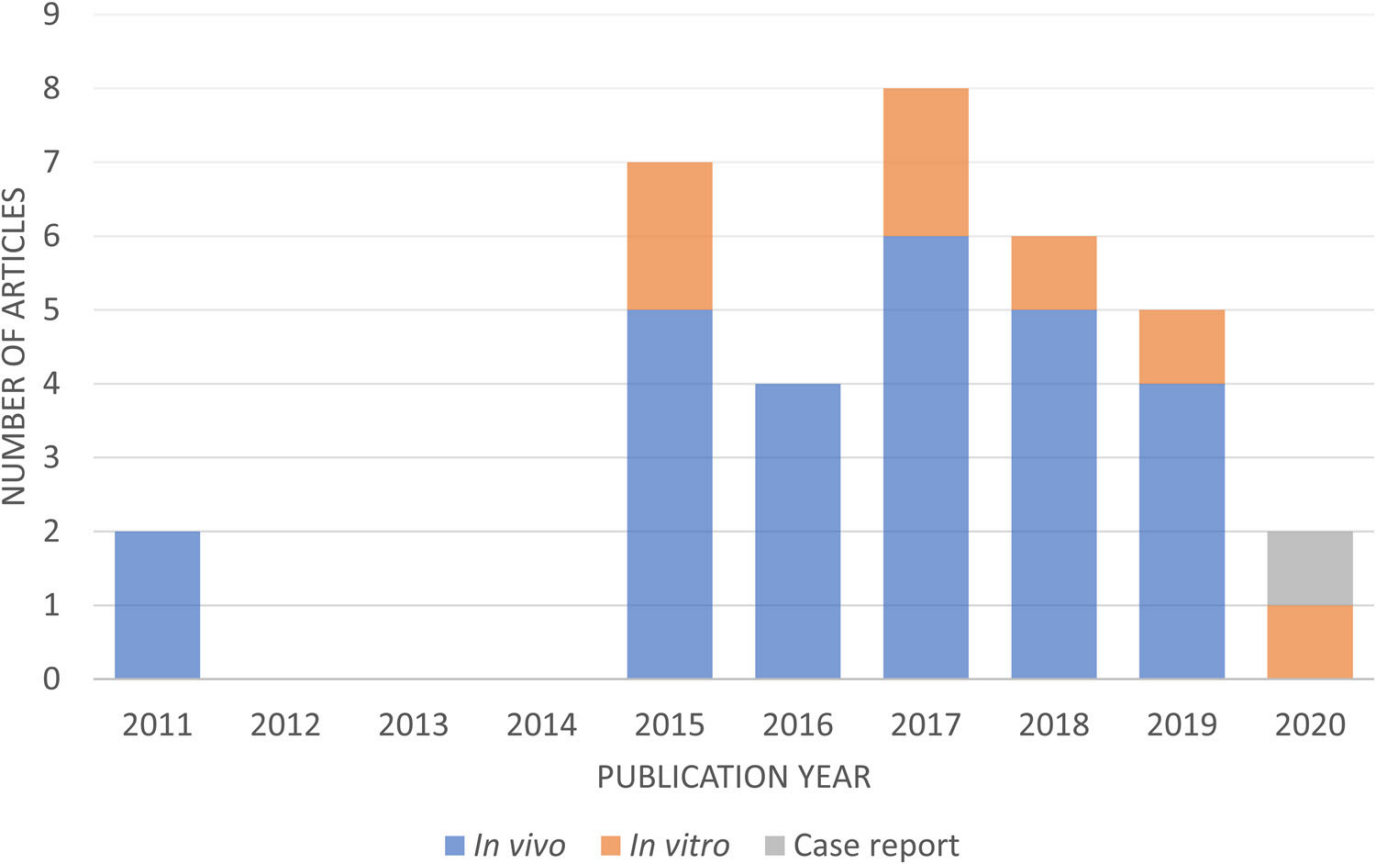
Temporal distribution of the number of articles published after 2010 which evaluate melatonin effects in hepatic fibrosis.

A general analysis of the studies published along time shows that the number of articles evaluating the beneficial effects of melatonin in hepatic fibrosis has been persistent and elevated in recent years (Figure 2). After the temporal gap observed between 2011 and 2015, the number of articles published markedly increased and remained high for several years, highlighting the interest in performing a complete review of all reported results to establish an overview in this regard. Curiously, previous literature screening showed the absence of studies conducted with human patients with liver fibrosis in which melatonin has been administered to evaluate its antifibrotic effects. Therefore, the present systematic review has been conducted only with preclinical studies.

**Figure 3:**
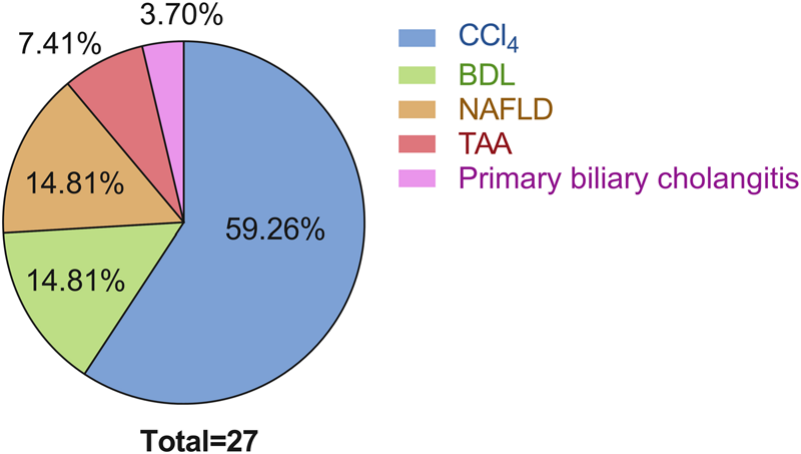
Representation of the percentage of articles that employed different methods for liver fibrosis induction. BDL, bile duct ligation; CCl_4_, carbon tetrachloride; NAFLD, nonalcoholic fatty liver disease; TAA, thioacetamide.

The main characteristics and data reported by the included articles are compiled in detail in Table 1. Among these studies, 2 articles employed only cell culture as a study model (6.90%), 22 used animal models (75.86%), and 5 used both in vitro and in vivo models (17.24%). Although there is a low number of articles with cell culture as the study model of hepatic fibrosis, a broad range of cell types have been employed, including H69, HepG2, LX2, and primary HSCs cell lines. Besides, a greater variety in time and concentration of melatonin treatment has been observed, ranging from 30 min to 21 days, and from 0.01 µM to 1 mM, respectively. Regarding in vivo models, the main method used to induce hepatic fibrosis was the intraperitoneal administration of CCl_4_, being also performed bile duct ligation (BDL), NAFLD induction by high‐fat diet (HFD), and primary biliary cholangitis, among others (Figure 3). Within them, 11 studies used mice and 16 rats as animal species for establishing the liver fibrosis model.

**Table 1:** Main characteristics of the included articles evaluating the melatonin effects on liver fibrosis and associated processes.

Research article	Method to liver failure	Experimental model	Administration strategy	Treatment regimen	General effects	Molecular alterations	
Ostrycharz et al. (2020)	Primary biliary cholangitis	In vitro	Melatonin	500 µM 24 h	Antiapoptotic	↓ PTEN	↓ p65
		− H69 cells^a^			Antioxidant	↑ Bcl‐2	↓ Nrf2
		− H69‐miR‐506 cells^b^			Anti‐inflammatory	↓ Bax	↑ miR‐132
		− Normal human cholangiocytes					
Liao et al. (2020)	CCl_4_	In vivo	ADSC pretreated with melatonin	10 μM	Higher graft efficiency		
		Male C57BL/6 mice					
D. J. Li et al. (2019)	NAFLD	In vivo	Intraperitoneal	30 mg/kg/day (8 weeks)	Antiapoptotic	↓ α‐SMA	↓ ASK1
		C57BL/6J mice	Melatonin			↓ TGF‐β	↓ Caspase‐3
		β‐arrestin‐1 knockout mice (Arrb1^−/−^)		10 μM 48 h	Antifibrotic	↓ pro‐col 1α1	↓ TUNEL‐positive cells
						↓ TNF‐α	
		*In vitro*				↓ IL‐1β	↓ p‐p38
		HepG2 with palmitic acid				↓ IL‐6	↓ p‐MKK3/6
Stacchiotti et al. (2019)	NAFLD	In vivo	Drinking water	10 mg/kg/day (16 weeks)	Ameliorated steatosis, ER stress, mitochondrial health and autophagy in HFD‐induced NAFLD, in WT but not in HET mice.	↓ GRP78	↑ SIRT1
		C57BL/6J mice (WT) and heterozygous SIRT1^+/−^ mice (HET)				↓ SREBP1	↓ p62
						↓ IL‐6	↓ miR‐34a‐5p
						↓ F4/80	↑ Mfn2
Haeger et al. (2019)	CCl_4_	In vivo	Drinking water	0.4 mg/kg/day (5 weeks)	Improvement cognitive behavior and		
		Sprague−Dawley male rats			motor skills		
Chen et al. (2019)	BDL + Pinealectomy	In vivo	Drinking water	2 mg/kg/day (1 week)	Antioxidant	↑ MT1	
		Male Fischer rats			Antifibrotic	↑ MT2	
					Antisenescence	↓ TGF‐β1	
					Regulation clock genes		
Bona et al. (2018)	CCl_4_ + Phenobarbital	In vivo	Intraperitoneal	20 mg/kg day (6 weeks)	Antifibrotic	↓ LPO	↓ iNOS
		Wistar rats			Antiangiogenic	↓ NQO1	↓ TGF‐β
					Anti‐inflammatory	↓ VEGF	↓ α‐SMA
						↓ p65	
Wang et al. (2018)	CCl_4_	In vivo	Intraperitoneal	2.5, 5.0 and 10.0 mg/kg/day for 6 weeks	Antioxidant	↓ MDA	↓ p‐Smad2/3
		Male Sprague−Dawley rats			Antifibrotic	↑ GPx	↓ TGF‐β1
						↓ Hyp	↑ Smad7
González‐Fernández et al. (2018)	CCl_4_	In vivo	Intraperitoneal	5 or 10 mg/kg/day for 2 or 4 weeks	Antifibrotic	↓ α‐SMA	↑ PER2
		Male C57BL/6 J mice					
		In vitro			Regulation clock genes	↓ Col1	↑ PER3
		LX2 cells				↑ PPARα	↑ CRY1
						↑ BMAL1	↑ CRY2
						↑ CLOCK	↓ REV‐ERBα
						↑ PER1	↑ RORα
Lebda et al. (2018)	TAA	In vivo	Intraperitoneal	5 mg/kg/day for a week before TAA, and 2 additional months	Antifibrotic	↓ MDA	↑ GST
		Male Wistar rats			Hepatoprotective	↓ TNF‐α	↑ PON‐1
						↓ IL‐1β	↓ Col1a
						↑ GSH	↓ Col3a
						↑ SOD	↓ TGF‐1β
						↑ CAT	↓ LN
						↑ GPx	↓ Enpp‐2
Mortezaee et al. (2018)	CCl_4_	In vivo	Intraperitoneal	20 mg/kg/day for a month	Antifibrotic	↓ Hyp	↑ MMP‐13
		Male Sprague−Dawley rats			Antioxidant	↑ Albumin	↓ TGF‐β1
						↑ SOD	↑ Bcl‐2
					Antiapoptotic	↑ GPx	↓ Bax
						↓ MDA	
McMillin et al. (2017)	BDL	In vivo	Intracerebroventricular	1 mg/kg/day for a week	Antifibrotic	↓ GnRH	↓ COL1A1
		Male Fischer rats				↓ CK19	↓ α‐SMA
		In vitro				↓ Ki67	↓ MMP‐9
		HSC cells					
Esrefoglu et al. (2017)	CCl_4_	In vivo	Intraperitoneal	10 mg/kg/day 24 h after CCl_4_ for 10 days	Antioxidant	↓ MDA	↑ GSH
		Female Wistar albino rats			Antiapoptotic	↑ SOD	↓ PCNA
						↑ CAT	
Wu et al. (2017)	Primary sclerosing cholangitis	In vivo	Drinking water	2 mg/g/day for a week	Antifibrotic	↓ PCNA	↓ Angpt1
		Male FVB/NJ wild‐type or Mdr2^−/−^ mice			Antiangiogenic	↓ COL1A1	↓ Angpt2
						↓ FN‐1	↓ Tie‐1
						↓ TGF‐1β	↓ Tie‐2
						↓ CK19	↓ CD31
						↓ VEGF‐A	↓ VWF
						↓ VEGF‐C	
Mortezaee et al. (2017)	CCl_4_	In vivo	Intravenous	MSCs incubated with 5 μM melatonin	Antifibrotic	↑ MMP‐9	↑ Bcl‐2
		Male Sprague−Dawley rats				↑ MMP‐13	↓ Bax
						↓ TGF‐β1	
Das et al. (2017)	HFD	In vivo	Intraperitoneal	10 and 20 mg/kg/day for 28 days	Antifibrotic	↓ α‐SMA	↑ MFN2
		Male C57BL/6 mice		1 mmol/L for 30 min	Antioxidant	↓ TGF‐β	↓ NOX4
		In vitro			Anti‐inflammatory		↓ FASN
		HepG2 cells					
						↓ Col1	↓ TNF‐α
						↓ TIMP1	↓ IL‐6
						↓ TIMP2	↓ Cleaved Caspase‐3
						↑ SIRT1	
González‐Fernández et al. (2017)	CCl_4_	In vivo	Intraperitoneal	5 or 10 mg/kg/day for 2 or 4 weeks	Antifibrogenic	↓ TGF‐β	↓ α‐SMA
						↓ Sphk1	↓ S1PR1
		Male C57BL/6 J mice				↓ Col1	↓ S1PR2
						↑ S1PL	↓ S1PR3
						↓ S1P	↓ ASMase
Colares et al. (2016)	BDL	In vivo	Intraperitoneal	20 mg/kg/day for 2 weeks	Antioxidant	↑ SOD	↓ GST
		Male Wistar rats			Anti‐inflammatory	↑ CAT	↓ iNOS
						↓ GPx	↓ TNF‐α
Nalobin et al. (2016)	CCl_4_	In vivo	Drinking water	20 μl/g for 7,14,21, or 30 days	Antifibrotic	↓ α‐SMA	
		C57Bl/CBA F1 hybrid mice aged					
Mortezaee et al. (2016)	CCl_4_	In vivo	Intravenous	MSCs incubated with 5 μM melatonin 24 h	Improvement of BMMSCs homing		
		Male Sprague−Dawley rats					
Kang et al. (2016)	CCl_4_	In vivo	Orally	2.5, 5, and 10 mg/kg/day	Mitophagy induction	↓ MDA	↑ Rab7
						↑ GSH/GSSG ratio	↑ LAMP2
		Male Sprague−Dawley rats			Mitochondrial protection		↑ PGC‐1α
						↑ SOD2	↑ Nrf1
						↑ PINK1	↑ TFAM
						↑ Parkin	↑ DRP1
						↓ LC3	↑ Mfn2
						↓ p62	↑ p‐AMPK
Czechowska et al. (2015)	TAA	In vivo	Intraperitoneal	10 mg/kg/day for 4 weeks	Anti‐inflammatory	↓ TNF‐α	↓ PDGF‐AB
						↓ IL‐6	↑ GSH
		Male Wistar rats			Antioxidant	↓ TGF‐β	↓ GSSG
					Antifibrotic	↓ IL‐1β	↑ PON‐1
Shajari et al. (2015)	HSCs	In vitro	Melatonin	10 µM for 4 h	Suppression of HSCs proliferation and activation	↓ Col1α1	↑ Nr1f1
		Primary rat HSCs				↓ Acta2	↓ Alox5
						↓ RORα	
San‐Miguel et al. (2015)	CCl_4_	In vivo	Intraperitoneal	5 or 10 mg/kg/day for 2 or 4 weeks	Antifibrogenic	↓ α‐SMA	↓ Atg12
		Male C57BL/6 J mice			Autophagy reduction	↓ LC3‐II	↓ Atg16L1
						↑ p62	↓ PERK
					ER stress suppression	↓ LAMP2	↓ ATF4
						↓ UVRAG	↓ ATF6
						↓ p‐mTOR	↓ IRE1
						↓ Beclin‐1	↓ XBP1S
						↓ Atg5	
Choi et al. (2015)	CCl_4_	In vivo	Orally	2.5, 5, and 10 mg/kg/day for 8 weeks	Antifibrotic	↓ caspase‐3	↓ RIP3
		Male Sprague−Dawley rats			Anti‐inflammatory	↓ TGF‐β	↓ MLKL
						↓ α‐SMA	↓ HMGB1
						↑ RIP1	↓ IL‐1α
Crespo et al. (2015)	CCl_4_	In vivo	Intraperitoneal	5 or 10 mg/kg/day for 2 or 4 weeks	Antifibrotic	↓ α‐SMA	↓ MMP‐9
		Male C57BL/6J mice				↓ Col I	↓ TIMP‐1
						↓ Col III	↓Amphiregulin
						↓ TGF‐β1	↓ p‐Smad3
						↓ PDGF	↑ Nrf2
						↓ CTGF	
Cho et al. (2015)	CCl_4_	In vivo	Intraperitoneal	5 mg/kg twice a week	Hepatic differentiation	↑ p‐ERK	↑ p‐IκB
		Male nude mice	Melatonin				
		In vitro		0.1–10 μM for 21 days	Anti‐inflammatory	↑ p‐JNK	↑ p65
		hDPSCs			Antifibrotic	↑ p‐p38	↑ NF‐κB
Renzi et al. (2011)	BDL	In vivo	Drinking water	2 mg/kg/day for 1 week	Antiproliferative	↓ CLOCK	↓ BMAL1
		Male Fischer rats			Antioxidant	↓ PER1	↓ PCNA
					Regulation clock genes	↓ CRY1	
Zaitone et al. (2011)	NAFLD	In vivo	Drinking water	10 mg/kg/day for 8 weeks	Antioxidant	↓ MDA	↑ GSH
		Male Wistar rats			Anti‐inflammatory	↓ TNF‐α	

Abbreviations: ADSC, adipose tissue‐derived mesenchymal stem cell; Alox5, Arachidonic Acid 5‐Lipoxygenase; AMPK, AMP‐activated protein kinase; Angpt, angiopoietin; ASK1, apoptosis signal‐regulating kinase 1; ASMase, acid sphingomyelinase; ATF, activating transcription factor; Atg, autophagy protein; BDL, bile duct ligation; BMAL1, brain and muscle Arnt‐like protein 1; BMMSCs, bone‐marrow‐derived mesenchymal stem cells; CAT, reduced catalase; CCl4, carbon tetrachloride; CK, cytokeratin; CLOCK, circadian locomotor output cycles kaput; Col, collagen; CRY, cryptochrome; CTGF, connective tissue growth factor; DRP1, dynamin‐related protein 1; Enpp‐2, Ectonucleotide pyrophosphatase/phosphodiesterase‐2; ER, endoplasmic reticulum; ERK1/2, extracellular regulated kinase 1/2; FASN, fatty acid synthase; FN1, fibronectin1; GnRH, gonadotrophin‐releasing hormone; GPx, glutathione peroxidase; GRP78, glucose regulated protein 78 kDa; GSH, reduced glutathione; GSSG, glutathione disulfide; GST, glutathione S‐transferase; hDPSCs, human dental pulp stem cells; HFD, high fat diet; HMGB1, high mobility group box 1 protein; HSCs, hepatic stellate cells; Hyp, hydroxyproline; IL, interleukin; iNOS, inducible nitric oxide synthase; IRE, inositol‐requiring enzyme; JNK, c‐Jun N‐terminal kinases; LAMP2, lysosome‐associated membrane glycoprotein 2; LC3, microtubule‐associated proteins 1A/1B light chain 3B; LN, laminin; LPO, lipid peroxidation; MDA, malondialdehyde; Mfn, mitofusin; MKK3/6, mitogen‐activated protein kinase kinase 3/6; MLKL, mixed lineage kinase domain‐like protein; MMP, metalloproteinase; MSCs, mesenchymal stem cells; MT, melatonin receptor; mTOR, mammalian target of rapamycin; NAFLD, nonalcoholic fatty liver disease; NF‐κB, nuclear factor‐κB; NOX, NADPH oxidase; NQO1, NAD(P)H:quinone oxidoreductase1; NRF1, nuclear respiratory factor 1; Nrf2, nuclear respiratory factor 2; PCNA, proliferating cell nuclear antigen; PDGF, platelet‐derived growth factor; PER, period circadian protein homolog; PERK, pancreatic ER kinase; PGC‐1α, peroxisome proliferator‐activated receptor‐gamma coactivator 1α; PINK1, PTEN‐induced putative kinase 1; PON, paraoxonase; PPARα, peroxisome proliferator‐activated receptor; PTEN, phosphatase and tensin homolog; Rab7, Ras‐related protein Rab‐7a; REV‐ERBα, nuclear receptor subfamily 1 group D1; RIP, receptor‐interacting protein; RORα, retinoic acid receptor‐related orphan receptor; S1P, sphingosine‐1 phosphate; S1PL, S1P lyase; S1PR, S1P receptor; SIRT1, sirtuin 1; SMA, smooth muscle actin; SOD, superoxide dismutase; Sphk1, sphingosine kinase 1; SQSTM1/p62, sequestosome 1; SREBP1, sterol regulatory element‐binding protein; TAA, thioacetamide; TFAM, transcription factor A mitochondrial; TGF, transforming growth factor; TIMP, tissue inhibitor of metalloproteinase; TNF, tumor necrosis factor; UVRAG, UV radiation resistance‐associated gene protein; VEGF, vascular endothelial growth factor; VWF, Von Willebrand factor; XBP1S, spliced X‐box‐binding protein‐1.

^a^ H69 cells: nontumor, SV40‐immortalized, human cholangiocytes.

^b^ H69‐miR‐506: H69 cells with experimental constitutive overexpression of miR‐506.

As the main treatment, melatonin was administered alone in all the included studies; however, only one of them evaluated the effects of combining melatonin with another compound (Zaitone et al., 2011). Moreover, the treatment strategy was different between studies, varying the dose from 0.4 to 30 mg/kg and the treatment duration from 1 to 16 weeks. Although several administration routes were used, intraperitoneal injection was the predominant (51.72%), followed by melatonin dissolved in drinking water (26.92%), intravenous injection (6.90%), oral (6.90%), injection of melatonin pretreated cells (6.90%), and intracerebroventricular administration (3.45%).

Numerous publications have employed in vivo and in vitro models to assess the role of melatonin in the progression of liver fibrosis in recent years. While an increasing number of studies have focused on antifibrotic properties of melatonin, a lower number of articles evaluated the melatonin effects on other processes and pathways related to hepatic fibrosis. Most included articles evaluated melatonin effects on oxidative stress and inflammation in liver fibrosis (46.43%), and a markedly small number of studies analyzed other processes underlying the beneficial effects of melatonin. Specifically, out of the 29 included articles, eight evaluated apoptosis (27.59%), three assessed autophagy and/or mitophagy (10.34%), circadian clocks (10.34%) and angiogenesis (10.34%), one analyzed the sphingosine kinase 1 (SphK1)/sphingosine‐1 phosphate (S1P) pathway (3.45%), two evaluated the role of microRNAs (6.90%) and one determined the alterations on behavior and cognitive skills (3.45%). The following section describes the main findings on mechanisms regulated by melatonin which are involved in hepatic fibrosis and associated cellular processes.

### 3.2 Role of melatonin in liver fibrosis

#### 3.2.1 Melatonin effects as an antifibrotic agent

The fibrotic state of the liver is mainly characterized by an activation of HSCs and collagen deposition, which leads to a progressive loss of liver functionality (Lee et al., 2015; Roehlen et al., 2020). Melatonin effects on liver fibrosis have been related to a decrease in different fibrotic and HSCs activation markers, including serum alanine aminotransferase (ALT) and aspartate aminotransferase (AST), alpha‐smooth muscle actin (α‐SMA), different collagen isoforms (Col1a1, Col3a1, ColI, ColIII), and collagen content in different in vivo models (Bona et al., 2018; Chen et al., 2019; Cho et al., 2015; Choi et al., 2015; Colares et al., 2016; Crespo et al., 2015; Czechowska et al., 2015; Das et al., 2017; González‐Fernández et al., 2017, 2018; Kang et al., 2016; Lebda et al., 2018; D. J. Li et al., 2019; McMillin et al., 2017; Mortezaee et al., 2017, 2018; Nalobin et al., 2016; San‐Miguel et al., 2015; Shajari et al., 2015; Wang et al., 2018; Wu et al., 2017; Zaitone et al., 2011), along with an increase in albumin levels observed in two studies performed with male Sprague−Dawley rats by the same research group (Mortezaee et al., 2017, 2018). These results were also observed when melatonin was combined with the insulin sensitizer pioglitazone in an in vivo model of NAFLD‐derived liver fibrosis (Zaitone et al., 2011).

During fibrogenesis, several cytokines are produced and secreted by liver cells, where transforming growth factor beta (TGF‐β) acts as one of the main profibrogenic cytokine (Roehlen et al., 2020). Different studies reported that melatonin administration either intraperitoneally, intravenously, orally, or in drinking water was able to reduce TGF‐β expression (Bona et al., 2018; Chen et al., 2019; Choi et al., 2015; Crespo et al., 2015; Czechowska et al., 2015; Das et al., 2017; González‐Fernández et al., 2017; Kang et al., 2016; Lebda et al., 2018; D. J. Li et al., 2019; Mortezaee et al., 2017, 2018; Wang et al., 2018; Wu et al., 2017). Decline in protein levels of Smad2/3 (Crespo et al., 2015; Wang et al., 2018), metalloproteinase‐9 (MMP‐9) (Crespo et al., 2015; McMillin et al., 2017), cyclic guanosine monophosphate, fibronectin‐1 (McMillin et al., 2017), platelet‐derived growth factor, connective tissue growth factor and tissue inhibitor of metalloproteinase 1 (Crespo et al., 2015) have also been shown, thus reducing HSCs activation. Similar results were found in the human HSCs cell line LX2 and primary HSCs, where melatonin decreased α‐SMA, TGF‐β, and COL1 expression (Das et al., 2017; Shajari et al., 2015). These antifibrotic effects have been also demonstrated treating bone‐marrow‐derived mesenchymal stem cells (BMMSCs) with 5 µM melatonin for 24 h and subsequently infiltrated through the tail vein in an animal model of CCl_4_‐induced fibrosis; this study showed a significant decrease in the fibrosis marker TGF‐β and in Masson's trichrome staining, as well as higher levels of MMP‐9 and MMP‐13 (Mortezaee et al., 2017). In another study performed with male Wistar rats, positive results on liver fibrosis after treatment with 20 mg/kg melatonin were also related to a decrease in hepatosomatic and splenosomatic indexes (HSI and SSI) (Colares et al., 2016). Curiously, pretreatment of adipose tissue‐derived mesenchymal stem cells (ADSC) with 10 µM melatonin also demonstrated greater therapeutic effects in a murine model of liver fibrosis. This was observed by a reduction of AST, ALT, and total bilirubin levels and lower fibrotic area in mice injected with melatonin‐treated ADSC through the tail vein (Liao et al., 2020). Along with these results, melatonin improved histological parameters distinctive of liver fibrosis. After inducing liver fibrosis through different mechanisms (CCl_4_ administration, HFD, and BDL) in mice and rat models, a variety of staining techniques including hematoxylin‐eosin, Masson's trichrome, and Sirius Red showed that melatonin administration diminished fibrotic area (Bona et al., 2018; Chen et al., 2019; Cho et al., 2015; Crespo et al., 2015; Das et al., 2017; Mortezaee et al., 2017; Zaitone et al., 2011). This was observed in some cases together with decreased hepatic steatosis (Das et al., 2017; Esrefoglu et al., 2017; Stacchiotti et al., 2019; Zaitone et al., 2011), lobular inflammation (Zaitone et al., 2011), intrahepatic bile duct mass (Chen et al., 2019; Wu et al., 2017), lipid peroxidation, and lipid content (Das et al., 2017).

Novel strategies to overcome fibrosis progression have been of recent interest, where cell therapy supposes a potential alternative to avoid whole organ allografts; however, there are still some drawbacks that need to be solved (Zhang et al., 2017). In this respect, a study conducted on male Sprague−Dawley rats with CCl_4_‐induced liver fibrosis demonstrated that BMMSCs pretreated with 5 µM melatonin favored the implantation BMMSCs in the liver parenchyma and had antifibrotic effects (Mortezaee et al., 2017). In addition to implantation‐associated problems, the lack of sources of embryonic and adult stem cells is also one of the main complications of cell therapy (Zhang et al., 2017). Research with human dental pulp stem cells (hDPSCs) also exhibited positive results when combined with melatonin; curiously, melatonin was able to induce hepatic differentiation of hDPSCs and reduce fibrotic markers levels, suppressing liver fibrosis and improving liver function (Cho et al., 2015).

**Figure 4:**
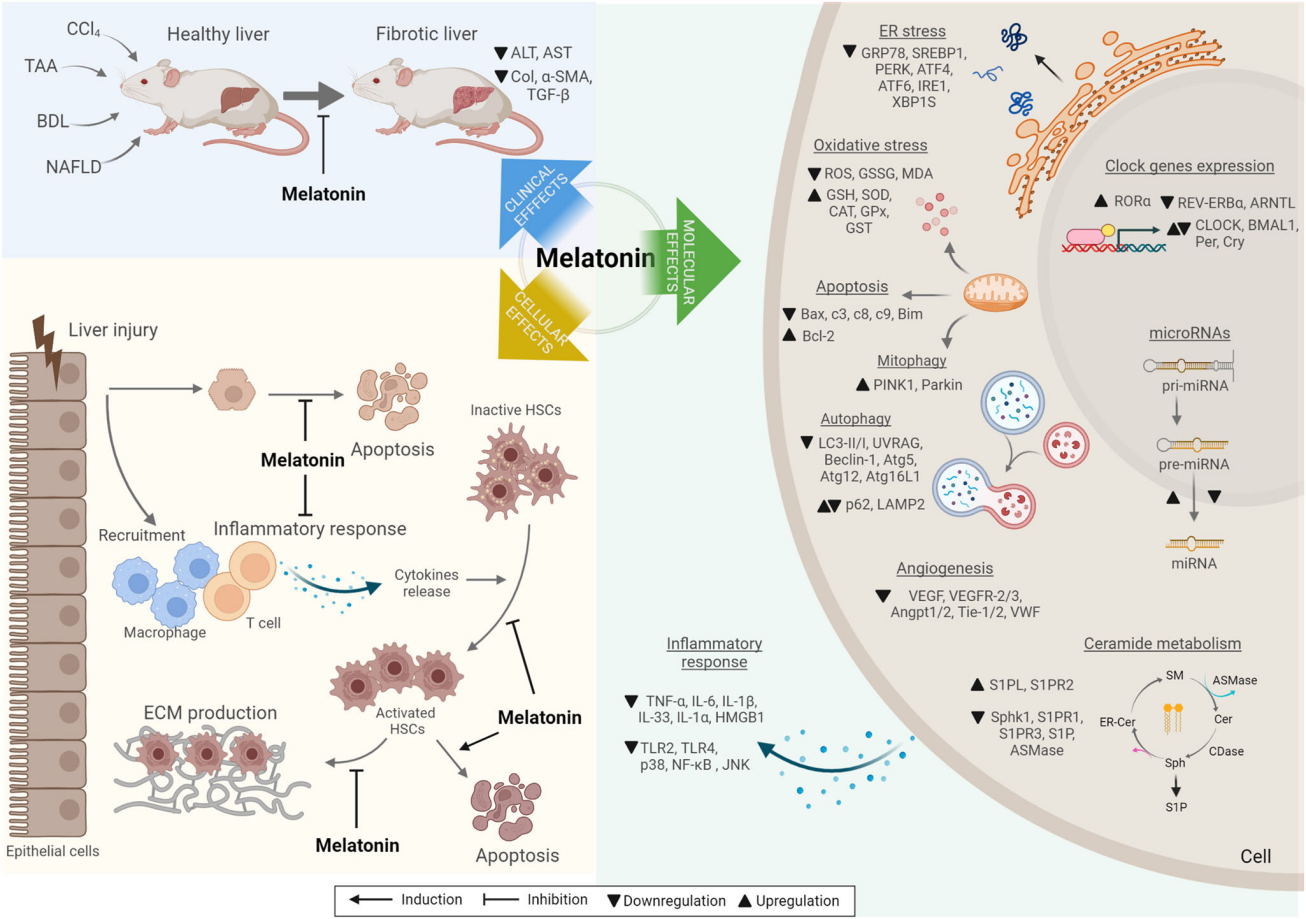
Representation of the main effects of melatonin observed in preclinical models of liver fibrosis. Melatonin administration is able to restrain fibrosis progression and reduce crucial profibrogenic and liver damage markers, such as α‐SMA, Col, TGF‐β, ALT, and AST. During fibrogenesis, several signaling pathways are deregulated, promoting the progression and establishment of hepatic fibrosis. Among them, ER stress, inflammatory response, circadian clock, oxidative stress, apoptosis, autophagy, mitophagy, angiogenesis, ceramide metabolism, and microRNAs seem to constitute key mechanisms that underlie the beneficial effects of melatonin on liver fibrosis. This indolamine has been demonstrated to modulate, upregulating or downregulating, the expression of several intermediates of the aforementioned processes, reversing the alterations triggered by fibrosis. Altogether, melatonin could act as an antifibrotic agent through modulation of key steps, both at clinical, cellular, and molecular levels, on liver fibrogenesis and fibrosis. α‐SMA, alpha‐smooth muscle actin; ALT, alanine aminotransferase; Angpt, angiopoietin; ARNTL, aryhydrocarbon receptor nuclear translocator‐like protein 1; ASMase, acid sphingomyelinase; AST, aspartate aminotransferase; ATF, activating transcription factor; Atg, autophagy protein; BDL, bile duct ligation; BMAL1, muscle Arnt‐like protein 1; c3, caspase‐3; c8, caspase‐8; c9, caspase‐9; CAT, catalase; CCl_4_, carbon tetrachloride; CDase, ceramidase; Cer, ceramide; CLOCK, circadian locomotor output cycles kaput; Col, collagen; Cry, cryptochrome; ECM, extracellular matrix; ER, endoplasmic reticulum; GPx, glutathione peroxidase; GRP78, glucose regulated protein 78 kDa; GSH, reduced glutathione; GSSG, glutathione disulfide; GST, glutathione S‐transferase; HMGB1, high mobility group box 1 protein; HSCs, hepatic stellate cells; IL, interleukin; IRE1, inositol‐requiring enzyme 1; LAMP2, lysosome‐associated membrane glycoprotein 2; MDA, malondialdehyde; miRNA, microRNA; NAFLD, nonalcoholic fatty liver disease; NF‐κB, nuclear factor‐κB; p62, sequestosome‐1; Per, period circadian protein homolog; PERK, pancreatic ER kinase; PINK1, PTEN‐induced putative kinase 1; REV‐ERBα, nuclear receptor subfamily 1 group D1; RORα, retinoic acid receptor‐related orphan receptor; ROS, reactive oxygen species; S1P, sphingosine‐1 phosphate; S1PL, S1P lyase; S1PR, S1P receptor; SM, sphingomyelin; SOD, superoxide dismutase; Sph, sphingosine; Sphk1, sphingosine kinase 1; SREBP1, sterol regulatory element‐binding protein; TAA, thioacetamide; TGF‐β, transforming growth factor beta; TNF‐α, tumor necrosis factor α; UVRAG, UV radiation resistance‐associated gene protein; VEGF, vascular endothelial growth factor; VEGFR, VEGF, vascular endothelial growth factor receptor; VWF, Von Willebrand factor; XBP1S, spliced X‐box‐binding protein‐1. This figure was created with BioRender.com.

The results here summarized demonstrated that melatonin acts as an antifibrotic agent, being able to modulate and restore cellular and tissue alterations caused by induction of liver fibrosis (Figure 4). Nonetheless, the study of the underlying mechanisms of these melatonin‐derived antifibrotic effects could suppose a potential tool to improve strategies against liver fibrosis. The main findings in this regard are summarized in the next sections.

#### 3.2.2 Antioxidant and anti‐inflammatory properties of melatonin in liver fibrosis

A great number of evidence have indicated that, besides etiology, oxidative stress and inflammation are the most relevant pathogenic events in liver diseases, including liver fibrosis (Reyes‐Gordillo et al., 2017). In addition to the antioxidant activity of melatonin widely described in several hepatic pathologies (Mortezaee & Khanlarkhani, 2018), the aforementioned antifibrotic effects observed in the liver by several investigations have been directly associated with melatonin antioxidant properties (Bona et al., 2018; Hu et al., 2016). In fact, because of its antioxidant activity, melatonin may face the onset and progression of inflammation (Mauriz et al., 2013).

Within the 29 included studies, four evaluated the melatonin‐derived effects only on the oxidative stress modulation, two on the inflammatory response, and seven assessed both antioxidant and anti‐inflammatory effects. Two different studies employed an animal model of thioacetamide (TAA)‐induced fibrosis with male Wistar rats. In both cases, liver fibrosis was manifested together with an increase in ROS production, that induced the expression of the inflammatory cytokines interleukin‐6 (IL‐6), IL‐1β, and tumor necrosis factor‐alpha (TNF‐α), and reduced catalase (CAT), glutathione peroxidase (GPx), glutathione S‐transferase (GST), superoxide dismutase (SOD), and reduced glutathione (GSH), core proteins of the antioxidant system that protects tissues against ROS generation (Czechowska et al., 2015; Lebda et al., 2018). Melatonin, as a direct scavenger of free radicals (Mauriz et al., 2013), abrogated these changes by reducing the hepatic oxidative stress indices and enhancing antioxidant enzyme activities (Czechowska et al., 2015; Lebda et al., 2018). Similarly, intraperitoneal administration of melatonin reversed DNA damage effects observed during liver fibrosis induction by decreasing malondialdehyde (MDA), protein carbonyl concentration, and DNA fragmentation (Lebda et al., 2018). Different research employing CCl_4_ as a fibrosis inductor also reported beneficial effects of melatonin against liver damage through antioxidant and anti‐inflammatory activities. Particularly, melatonin intraperitoneally and orally administered increased GPx, SOD, CAT, and GSH production, decreased MDA and inducible nitric oxide synthase (iNOS) expression (Bona et al., 2018; Esrefoglu et al., 2017; Kang et al., 2016; Mortezaee et al., 2018; Wang et al., 2018) and suppressed the inflammatory response by reducing the expression of Toll‐like receptors 2 and 4 (TLR2 and TLR4), serum high mobility group box 1 protein (HMGB1), IL‐1α, and phosphorylation of JNK, p38, nuclear factor‐κB (NF‐κB) (Bona et al., 2018; Choi et al., 2015; Kang et al., 2016). Contrariwise, no effects were observed on IL‐33 expression and phosphorylation of ERK after melatonin treatment (Choi et al., 2015). Surgical induction and NAFLD‐derived liver fibrosis have been also used for evaluating melatonin modulation ability on the antioxidant and anti‐inflammatory responses (Chen et al., 2019; Colares et al., 2016; Das et al., 2017; D. J. Li et al., 2019; Zaitone et al., 2011). After inducing liver fibrosis, melatonin restored the redox balance by decreasing the levels of ROS, NADPH oxidase 4 (NOX4), iNOS, and MDA, and by rising the activity of several antioxidant enzymes, and the expression of SOD, CAT, and GSH (Chen et al., 2019; Colares et al., 2016; Das et al., 2017; Zaitone et al., 2011). Curiously, there are some opposite results where the levels of GPx, GST, and GSH were lower after melatonin administration via intraperitoneal in male Wistar rats with liver fibrosis induced by BDL (Colares et al., 2016). Nonetheless, all the studies that evaluated melatonin effects on inflammation found an anti‐inflammatory activity represented by declined levels of TNF‐α, IL‐6, IL‐1β, and IL‐33 (Chen et al., 2019; Colares et al., 2016; Das et al., 2017; D. J. Li et al., 2019; Zaitone et al., 2011). A study also analyzed these effects when combining melatonin with pioglitazone, showing again a reduction of the oxidative stress and inflammatory response after the treatment combination (Zaitone et al., 2011). Recent research conducted in a murine model of liver fibrosis has demonstrated that antifibrotic effects of melatonin are associated with the inhibition of apoptosis signal‐regulating kinase 1 (ASK1) activation by promoting its degradation in the liver through the blockage of TNF receptor‐associated factors (TRAFs)‐ASK1 interaction in response to oxidative stress and proinflammatory stimuli (D. J. Li et al., 2019).

Chronic liver injury that triggers a fibrotic state of the liver is closely related to an increase in oxidative stress and to an inflammatory response, promoting liver fibrosis progression. Results reported by different studies indicate that melatonin seems to act as a potent antioxidant and modulator of the inflammatory response as part of its antifibrotic activity in liver fibrosis (Figure 4).

#### 3.2.3 Modulation of apoptosis and autophagy by melatonin in liver fibrosis

Hepatocyte death plays a key role in the progression of liver pathologies. During liver fibrosis, injured hepatic cells undergoing apoptosis cell death promote and trigger a profibrogenic response (Roehlen et al., 2020). Cell apoptosis is a type of cell death characterized by cell shrinkage, chromatin remodeling, and the formation of apoptotic bodies (Barangi et al., 2020; Roehlen et al., 2020). Released DNA and apoptotic bodies from hepatocytes can be phagocytosed by Kupffer cells and HSCs, leading to cell activation and fibrosis progression (Roehlen et al., 2020).

As represented in Figure 4, several studies have found that fibrosis induction in the liver of either rats or mice promoted an increase in liver cell apoptosis (Choi et al., 2015; Esrefoglu et al., 2017; D. J. Li et al., 2019; Mortezaee et al., 2017, 2018; Renzi et al., 2011). Results indicated that melatonin administration was able to decrease positive apoptotic cells (Choi et al., 2015; Esrefoglu et al., 2017), Bax expression (Mortezaee et al., 2017, 2018), and cleaved caspase‐3/caspase‐3 ratio (Choi et al., 2015), and induce higher levels of the antiapoptotic protein Bcl‐2 (Mortezaee et al., 2017, 2018), using different animal models with CCl_4_‐derived liver fibrosis. Similarly, apoptosis induced after BDL and NAFLD‐derived fibrosis was prevented when melatonin was administered by intraperitoneal injection or drinking water, respectively (D. J. Li et al., 2019; Renzi et al., 2011). Antiapoptotic effects of the indolamine related to its antifibrotic activity have been also observed in vitro, where human H69 cholangiocytes subjected to oxidative stress experienced an increase in the apoptotic markers phosphatase and tensin homolog (PTEN), Bax, cleaved caspase‐3 and Bim, and a reduction of Bcl‐2. Results showed that melatonin was achieved to protect cholangiocytes against apoptosis and these effects were mediated by an increase in the expression of the microRNA miR‐132 and a decrease of miR‐34 (Ostrycharz et al., 2020). In addition, melatonin also modulated apoptosis in a coculture of hepatocytes (HepG2) and HSCs (LX2), but in a different way since it induced LX2 apoptosis thus preventing HSCs activation (Das et al., 2017). Necroptosis, a new mechanism of cell death similar to apoptosis, has been also suggested to be involved in liver fibrosis (Choi et al., 2015). An in vivo study with CCl_4_ as fibrosis inductor in rats determined that, even though the necroptotic marker receptor‐interacting protein 1 (RIP1) was not modified in any experimental group, RIP3 and mixed lineage kinase domain‐like protein (MLKL) expression increased significantly after hepatic fibrosis establishment. Likewise, melatonin administration attenuated all these increases, suggesting that indolamine is also able to inhibit the necroptosis signaling pathway during liver fibrosis (Choi et al., 2015).

Autophagy, a catabolic and self‐recycling process, is an important modulator of liver homeostasis under both physiological and pathological conditions (Mallat et al., 2014). The role of autophagy in liver fibrosis is still controversial and both prosurvival and proapoptotic effects have been described (Kang et al., 2016; San‐Miguel et al., 2015). Despite autophagy has been closely related to several liver pathologies, only three out of 29 articles included in this systematic review evaluated melatonin effects on autophagy associated with its antifibrotic properties in the liver (Kang et al., 2016; San‐Miguel et al., 2015; Stacchiotti et al., 2019).

One of the studies conducted by our group found that melatonin administration restrained autophagy induction in liver fibrosis by decreasing the number of autophagosomes, the LC3‐II/I ratio, and the expression of lysosome‐associated membrane glycoprotein 2 (LAMP2), UV radiation resistance‐associated gene protein (UVRAG), phosphorylated mammalian target of rapamycin (p‐mTOR), Beclin‐1, autophagy protein 5 (Atg5), Atg12, Atg16L1, and by increasing the levels of sequestosome‐1 (SQSTM1/p62) (San‐Miguel et al., 2015). Along with these results, melatonin was also able to reduce the expression of endoplasmic reticulum (ER) stress markers, such as pancreatic ER kinase (PERK), activating transcription factor 4 (ATF4), ATF6, inositol‐requiring enzyme 1 (IRE1) and spliced X‐box‐binding protein‐1 (XBP1S) (San‐Miguel et al., 2015). Conversely, a research group that analyzed melatonin properties in NAFLD‐derived fibrosis reported a raise in autophagosomes, mitofusin 2 (Mfn2), and a decrease in cytoplasmic SQSTM1/p62 levels. Nevertheless, melatonin effects on ER stress were also determined and similar to those previously described by our group, finding a reduction in glucose‐regulated protein 78 kDa (GRP78) and sterol regulatory element‐binding protein (SREBP1) expression (Stacchiotti et al., 2019). Mitophagy, a specific type of autophagy, is responsible for removing and recycling damaged mitochondria in cells and could have a key role in maintaining cellular homeostasis (Mallat et al., 2014). Melatonin modulation of mitophagy and mitochondria homeostasis in liver fibrosis was evaluated in an animal model of CCl_4_‐induced fibrosis. Results showed an increase in the levels of several mitophagy and mitochondria biogenesis markers, including PTEN‐induced putative kinase 1 (PINK1), Parkin, Ras‐related protein Rab‐7a (Rab7), LAMP2, peroxisome proliferator‐activated receptor‐gamma coactivator 1α (PGC‐1α), nuclear respiratory factor 1 (NRF1), transcription factor A mitochondrial (TFAM), dynamin‐related protein 1 (DRP1) and Mfn2 (Kang et al., 2016). Along with this, melatonin raised the number of autophagy vacuoles and the amount of mitochondrial DNA, exhibiting a mitophagy inductor role (Kang et al., 2016). Melatonin has also been shown to ameliorate mitochondrial dysfunction through Sirtuin 1 (SIRT1) activation, a key factor involved in mitochondrial morphology maintenance, in a mitophagy‐independent way (Das et al., 2017). Melatonin repressed cytokines and ROS, responsible for initiating mitochondrial dysfunction in hepatocytes and, as consequence, prevented HSCs activation and hindered the progression of liver fibrosis (Das et al., 2017).

Altogether, melatonin has shown to exert different activities on apoptosis and autophagy as liver fibrosis‐associated processes, thus modulating cell response to hepatic damage and liver fibrosis progression (Figure 4).

#### 3.2.4 Melatonin effects in other cellular processes

Together with the above‐mentioned alterations, some studies have described several processes and mechanisms that could mediate melatonin‐derived effects on liver fibrosis.

##### Circadian clock

Dysregulation of the circadian clock machinery is a critical mechanism in the pathogenesis of fibrosis (González‐Fernández et al., 2018). Despite melatonin is a widely known hormone with chronobiotic activity related to its beneficial properties against several human pathologies (Mauriz et al., 2013), only three out of 29 articles analyzed the modulatory actions of melatonin in the clock machinery (Chen et al., 2019; González‐Fernández et al., 2018; Renzi et al., 2011) (see Figure 4). One study has shown that the decrease in melatonin induced by prolonged exposure to light or after pinealectomy increased liver fibrosis and deregulated circadian clock genes expression (Chen et al., 2019). Lack of melatonin production and secretion led to higher levels of circadian locomotor output cycles kaput (CLOCK), aryhydrocarbon receptor nuclear translocator‐like protein 1 (ARNTL), cryptochrome 1 (Cry1), and period circadian protein homolog 1 (Per1) in both total liver and cholangiocytes (Chen et al., 2019). Similarly, melatonin administration by drinking water to male Fischer rats subjected to BDL promoted a decrease in several clock genes expression, including CLOCK, Per1, Cry1, and brain and muscle Arnt‐like protein 1 (BMAL1), along with reduced levels of the melatonin receptors MT1 and MT2 (Renzi et al., 2011). However, since circadian rhythms act as a dynamic and context‐dependent machinery, opposite results have been reported regarding melatonin modulation activity. Data obtained by our group demonstrated that the establishment of liver fibrosis by CCl_4_ in a murine model deregulated circadian clock by diminishing BMAL1, CLOCK, Per1, Per2, Per3, Cry1, Cry2, and retinoic acid receptor‐related orphan receptor (RORα) and increasing nuclear receptor subfamily 1 group D1 (REV‐ERBα) levels (González‐Fernández et al., 2018). Intraperitoneal administration of melatonin was able to prevent all the alterations derived from fibrosis induction and restore clock genes expression in a dose‐dependent manner (González‐Fernández et al., 2018). These results were also reproduced in LX2 cells, where the indolamine also induced a dose‐dependent increase in the expression of BMAL1, CLOCK, Per2, Cry1, and RORα, and a decrease of REV‐ERBα (González‐Fernández et al., 2018). Although opposite results have been found regarding circadian rhythms, all the studies suggest that the regulation of circadian clocks exerted by melatonin both in vitro and in vivo may contribute to attenuate liver fibrosis (Figure 4).

##### Sphingosine signaling

Within the broad number of signaling pathways involved in liver pathologies, the interrelationship between the SphK1/S1P system and liver fibrosis has been also studied (González‐Fernández et al., 2017) (Figure 4). Chronic CCl_4_ injury increased SphK1 expression as well as S1P production, but in contrast, S1P lyase (S1PL) was markedly decreased. These effects were significantly abrogated by melatonin in a dose‐dependent manner, suggesting that the SphK1/S1P pathway could be involved in the antifibrotic effect exerted by melatonin (González‐Fernández et al., 2017). Similar results were obtained in an in vitro cell model where melatonin reduced the levels of the main fibrosis markers along with an inhibition of the sphingosine pathway axis (González‐Fernández et al., 2017).

##### Angiogenesis

Liver fibrosis has been related to changes at the vascular level, developing a hypoxic environment that promotes the generation of new blood vessels, a process known as angiogenesis (Bona et al., 2018). However, a low number of studies have focused on angiogenesis modulation as a constitutive mechanism of liver fibrosis. One of the studies that assessed melatonin actions on angiogenesis employed an in vivo model of cholangitis‐derived fibrosis; results indicated that melatonin prevented the increase of vascular endothelial growth factor receptor 2 (VEGFR‐2), VEGFR‐3, VEGF, angiopoietin 1 and 2, Tie‐1, Tie‐2 and Von Willebrand factor (VWF) expression (Wu et al., 2017). These effects were also observed in a CCl_4_ fibrotic model, where intraperitoneal melatonin diminished VEGF levels (Bona et al., 2018). Reduction of melatonin production by prolonged light exposure or pinealectomy showed an increase in VEGFA, VWF, and platelet endothelial cell adhesion molecule 1 (PECAM‐1) (Chen et al., 2019), suggesting an interesting role of the indole through angiogenesis modulation. In this study, the authors also found that the absence of melatonin synthesis enhanced the expression of biliary senescence markers, including p16, p21, C‐C motif chemokine ligand 2 (CCL2) and senescence‐associated β galactosidase (SA‐β‐gal), leading to hepatic fibrosis progression (Chen et al., 2019) (Figure 4).

##### MicroRNAs

MicroRNAs (miRNAs) are small, noncoding RNAs that are frequently deregulated in liver diseases and have acquired an important interest recently, emerging as new crucial regulators of cellular processes (Jiang et al., 2017)_._ As represented in Figure 4, some relevant results have been obtained from an in vivo and in vitro study that employed cholangiocytes from WT and Mdr^2/2^ mice exposed to dark and supplemented with melatonin in drinking water. Moreover, the effect of melatonin in cholangiocytes and HSCs isolated from WT and Mdr^2/2^ after the inhibition of miR‐200b was also tested. Hepatic fibrosis was significantly reduced by dark exposure or melatonin treatment compared with WT mice, finding that the inhibition of miR‐200b expression induced by melatonin treatment or prolonged darkness was involved in its observed antifibrotic properties (Wu et al., 2017). Similarly, research carried out in a NAFLD‐derived fibrosis model reported that melatonin restored Sirtuin1 (SIRT1) levels, protein responsible for regulating longevity and cellular metabolism, by inhibiting miR‐34a‐5p expression. The beneficial role of melatonin observed in fibrotic mice was linked to the lower levels of miR‐34a‐5p, demonstrating the protective role of melatonin against liver fibrosis (Stacchiotti et al., 2019).

##### Behavior and cognition

Curiously, a study employed male Sprague−Dawley rats with CCl_4_‐induced liver fibrosis to evaluate the alterations on behavior and cognitive skills. Higher escape latency and speed were observed when fibrosis was induced, while melatonin administration reduced these parameters, improving the motor skills and cognition of fibrotic rats (Haeger et al., 2019).

## 4 DISCUSSION

The present systematic review aimed to identify and compile the main effects of melatonin as an antifibrotic agent in liver fibrosis. Included articles described a broad diversity of molecular aspects improved by melatonin administration that were associated to its antifibrotic effects (Figure 4). These studies used a wide range of melatonin concentrations and administration strategies, which highlights the need for higher consistency to provide translational results to human patients. Remarkably, exogenous administration to human subjects of different melatonin concentrations has been tested in several clinical trials. A study compiling main findings of these trials reported that doses from 2 to 500 mg/day show positive but not toxic effects caused by melatonin, even with the highest concentration (Reiter et al., 2022). Regarding animal models, the lethal dose for 50% of the subjects (LD50) could not be established, even administrating melatonin at 800 mg/kg (Malhotra et al., 2004). When endogenous melatonin is evaluated, normal circulating plasma levels in humans have been found to be up to 60 pg/ml as the peak level (Kennaway, 2020). Although no melatonin measurements in plasma samples have been performed in the included studies, the highest dose employed of melatonin was 30 mg/kg (D. J. Li et al., 2019). Despite the differences in administration route, dosage, and method of fibrosis induction, melatonin is demonstrated to restrain hepatic fibrosis, improving fibrotic markers and histopathology of animal livers (Bona et al., 2018; Chen et al., 2019; Cho et al., 2015; Choi et al., 2015; Colares et al., 2016; Crespo et al., 2015; Czechowska et al., 2015; Das et al., 2017; Esrefoglu et al., 2017; González‐Fernández et al., 2017, 2018; Haeger et al., 2019; Kang et al., 2016; Lebda et al., 2018; D. J. Li et al., 2019; Liao et al., 2020; McMillin et al., 2017; Mortezaee et al., 2017, 2018; Nalobin et al., 2016; Ostrycharz et al., 2020; Renzi et al., 2011; San‐Miguel et al., 2015; Shajari et al., 2015; Stacchiotti et al., 2019; Wang et al., 2018; Wu et al., 2017; Zaitone et al., 2011). In line with this, beneficial properties of melatonin have been broadly reported in several liver pathologies, such as FHF (Laliena et al., 2012) and HCC (Carbajo‐Pescador et al., 2013; Sánchez et al., 2018). Notwithstanding, there is a lack of investigations that analyze the molecular aspects underlying these antifibrotic effects of melatonin.

Loss of redox balance together with a proinflammatory response constitute two of the main factors associated with liver fibrosis induction and progression (Reyes‐Gordillo et al., 2017). Most articles included in this systematic review have reported the antioxidant and anti‐inflammatory actions of melatonin as part of its antifibrotic effects. Similarly, several investigations carried out with different hepatic disease models have also reported antioxidant properties of melatonin directly related to its positive activity on abrogating NAFLD and hepatic ischemic injury, among others (Bosco et al., 2019; Joshi et al., 2021; H. W. Li et al., 2021). Together with oxidative stress, inflammation is a process highly associated with hepatic damage (Mortezaee & Khanlarkhani, 2018), where melatonin has also been demonstrated to modulate the inflammatory response in several liver pathologies by restraining an overactivated response derived from chronic liver injury (Laliena et al., 2012; H. W. Li et al., 2021) or even from hepatotoxicity (Yang et al., 2021). Considering the results summarized in the present systematic review (see Figure 4), melatonin could potentially act as an effective antifibrotic agent through its antioxidant and anti‐inflammatory properties.

Otherwise, a small number of studies analyzed some processes underlying the beneficial effects of melatonin on liver fibrosis, such as apoptosis, autophagy, and circadian clocks, among others (Figure 4). Melatonin administration has proved to modulate both apoptosis and autophagy in a cell and context‐dependent manner, by promoting HSCs apoptosis and restraining hepatocyte apoptosis (Choi et al., 2015; Das et al., 2017; Esrefoglu et al., 2017; D. J. Li et al., 2019; Mortezaee et al., 2018; Ostrycharz et al., 2020; Renzi et al., 2011); and by either inhibiting or inducing autophagy (Kang et al., 2016; San‐Miguel et al., 2015; Stacchiotti et al., 2019). This is in line with melatonin‐derived changes in apoptotic and autophagic markers, which were decreased in different hepatic pathologies caused by liver injury, liver ischemia, and hepatotoxicity (Barangi et al., 2020; Kang et al., 2014; H. W. Li et al., 2021; Yang et al., 2021). Likewise, contrary results were also observed in benzo(a)pyrene‐injured liver and HCC, where melatonin promoted autophagy and apoptosis of tumoral hepatocytes, respectively (Barangi et al., 2020; Carbajo‐Pescador et al., 2013; El‐Magd et al., 2019). These results suggest that melatonin‐associated effects on apoptosis and autophagy could be directly related to its antifibrotic activity (Figure 4). Nonetheless, the low number of conducted studies and the differential role played by melatonin raise the need for further analysis to accurately determine the role of apoptosis and autophagy modulation.

Circadian clock has a key role in liver physiology and its alteration is known to favor fibrosis (González‐Fernández et al., 2018; Joshi et al., 2021), where melatonin has shown to restore the circadian machinery altered during fibrosis progression in results from included studies (Figure 4) (Chen et al., 2019; González‐Fernández et al., 2018; Renzi et al., 2011). Beneficial effects of this indole in reestablishing circadian rhythms have been also observed in other liver pathologies, including NAFLD (Joshi et al., 2021) and HCC (Sánchez et al., 2018).

Regarding signaling pathways, published articles have focused only on vascular‐related pathways and lipid metabolism (see Figure 4). Changes due to melatonin administration have been briefly assessed, showing an antiangiogenic action of the indolamine (Chen et al., 2019; Wu et al., 2017) as well as inhibition of the Sphk1/S1P pathway (González‐Fernández et al., 2017). A blockage in the sphingosine‐related pathways due to melatonin administration has also been reported in FHF, associated with other melatonin protective actions in the liver (Crespo et al., 2016). Contrarily, despite the broadly described antiangiogenic role of melatonin in liver cancer (El‐Magd et al., 2019), there are no studies performed on other liver pathologies.

Finally, as shown in Figure 4, some evidence have associated melatonin actions against hepatic fibrosis with alterations in the expression and function of different miRNAs (Stacchiotti et al., 2019; Wu et al., 2017). Synergistic effects exerted by miRNA, either alone or combined, have been related to HSCs activation as well as to the progression of liver fibrosis through interaction with molecules derived from different signaling pathways (Jiang et al., 2017). Similarly, melatonin has also demonstrated to prevent benzo(a)pyrene hepatotoxicity through modulation of the miR‐34a/SIRT1/autophagy axis (Barangi et al., 2020).

However, despite the crucial role that melatonin seems to exert in circadian rhythms maintenance, as well as in key signaling pathways and microRNAs modulation, as part of its beneficial role in liver fibrosis, additional studies need to be performed to clearly determine the exact mechanisms of action.

### 4.1 Limitations

The main limitations found in this systematic review are due to the high variation in the study design. As mentioned in the results section, the range of melatonin dosages used is wide, ranking from µM to mM units, and treatment duration differs from minutes to several days. Although the melatonin concentrations used in the included studies are within safe ranges, higher consistency in dose selection across studies would be of greatest interest, together with clinical analysis of plasma levels of melatonin in the animal models as part of the study design to establish safe dosing ranges. Moreover, up to six different administration routes of melatonin were employed. These high variations in study design become a relevant limitation and hinder the correct understanding of melatonin's effects on liver fibrosis. Although the variety in the methods used for fibrosis induction could also suppose a limitation, more than a half of the studies employed the same method, improving the consistency in the study design. Finally, melatonin effects were evaluated mainly as a single treatment, with only one publication in which a potential combination of melatonin with other compounds was analyzed in liver fibrosis.

## 5 CONCLUSION AND FUTURE PERSPECTIVES

The present study constitutes the first systematic review in which melatonin effects on liver fibrosis have been deeply described. Biological evidence reported in the last years regarding cellular and molecular mechanisms of melatonin antifibrotic actions has been reviewed in detail. A large number of articles have demonstrated the beneficial effects of melatonin administration on liver fibrosis through the modulation of several mechanisms and processes (oxidative stress, inflammation, apoptosis, autophagy, circadian clocks, among others). However, further studies are needed to fully determine and clarify the exact mechanisms that mediate the protective effects of melatonin, since novel findings could improve the clinical onset of human patients with not only hepatic fibrosis but also different liver pathologies.

## AUTHOR CONTRIBUTIONS

All authors were responsible for study conception and design, interpretation of the data, and drafting of the manuscript. Systematic literature review, data extraction, and data analysis were performed by Beatriz San‐Miguel and Paula Fernández‐Palanca. Moreover, José L. Mauriz, María J. Tuñón, and Javier González‐Gallego carried out the study supervision, review and editing of the paper. The final version of the manuscript was approved by all authors.

## CONFLICTS OF INTEREST

The authors declare no conflicts of interest.

## Supporting information

Supplementary information.

## References

[jcp30735-bib-0001] Barangi, S. , Mehri, S. , Moosavi, Z. , Hayesd, A. W. , Reiter, R. J. , Cardinali, D. P. , & Karimi, G. (2020). Melatonin inhibits Benzo(a)pyrene‐Induced apoptosis through activation of the Mir‐34a/Sirt1/autophagy pathway in mouse liver. Ecotoxicology and Environmental Safety, 196(March), 110556. 10.1016/j.ecoenv.2020.110556 32247962

[jcp30735-bib-0002] Bona, S. , Rodrigues, G. , Moreira, A. J. , Di Naso, F. C. , Dias, A. S. , Da Silveira, T. R. , Marroni, C. A. , & Marroni, N. P. (2018). Antifibrogenic effect of melatonin in rats with experimental liver cirrhosis induced by carbon tetrachloride. JGH Open, 2(4), 117–123. 10.1002/jgh3.12055 30483575 PMC6206983

[jcp30735-bib-0003] Bosco, A. D. , Schedler, F. B. , Colares, J. R. , Schemitt, E. G. , Hartmann, R. M. , Forgiarini Junior, L. A. , Dias, A. S. , & Marroni, N. P. (2019). Melatonin effects on pulmonary tissue in the experimental model of hepatopulmonary syndrome. Jornal Brasileiro de Pneumologia, 45(3), e20170164. 10.1590/1806-3713/e20170164 31166552 PMC6715043

[jcp30735-bib-0004] Carbajo‐Pescador, S. , Steinmetz, C. , Kashyap, A. , Lorenz, S. , Mauriz, J. L. , Heise, M. , Galle, P. R. , González‐Gallego, J. , & Strand, S. (2013). Melatonin induces transcriptional regulation of Bim by FoxO3a in HepG2 cells. British Journal of Cancer, 108(2), 442–449. 10.1038/bjc.2012.563 23257900 PMC3566813

[jcp30735-bib-0005] Chen, L. , Zhou, T. , Wu, N. , O'Brien, A. , Venter, J. , Ceci, L. , Kyritsi, K. , Onori, P. , Gaudio, E. , Sybenga, A. , Xie, L. , Wu, C. , Fabris, L. , Invernizzi, P. , Zawieja, D. , Liangpunsakul, S. , Meng, F. , Francis, H. , Alpini, G. , … Glaser, S. (2019). Pinealectomy or light exposure exacerbates biliary damage and liver fibrosis in cholestatic rats through decreased melatonin synthesis. Biochimica et Biophysica Acta—Molecular Basis of Disease, 1865(6), 1525–1539. 10.1016/j.bbadis.2019.03.002 30890428 PMC6993622

[jcp30735-bib-0006] Cho, Y. A. , Noh, K. , Jue, S. S. , Lee, S. Y. , & Kim, E. C. (2015). Melatonin promotes hepatic differentiation of human dental pulp stem cells: Clinical implications for the prevention of liver fibrosis. Journal of Pineal Research, 58(1), 127–135. 10.1111/jpi.12198 25431168

[jcp30735-bib-0007] Choi, H. S. , Kang, J. W. , & Lee, S. M. (2015). Melatonin attenuates carbon tetrachloride‐induced liver fibrosis via inhibition of necroptosis. Translational Research, 166(3), 292–303. 10.1016/j.trsl.2015.04.002 25936762

[jcp30735-bib-0008] Colares, J. R. , Schemitt, E. G. , Hartmann, R. M. , Licks, F. , Do Couto Soares, M. , Dal Bosco, A. , & Marroni, N. P. (2016). Antioxidant and anti‐inflammatory action of melatonin in an experimental model of secondary biliary cirrhosis induced by bile duct ligation. World Journal of Gastroenterology, 22(40), 8918–8928. 10.3748/wjg.v22.i40.8918 27833383 PMC5083797

[jcp30735-bib-0009] Crespo, I. , San‐Miguel, B. , Fernández, A. , Ortiz De Urbina, J. , González‐Gallego, J. , & Tuñón, M. J. (2015). Melatonin limits the expression of profibrogenic genes and ameliorates the progression of hepatic fibrosis in mice. Translational Research, 165(2), 346–357. 10.1016/j.trsl.2014.10.003 25445210

[jcp30735-bib-0010] Crespo, I. , San‐Miguel, B. , Sánchez, D. I. , González‐Fernández, B. , Álvarez, M. , González‐Gallego, J. , & Tuñón, M. J. (2016). Melatonin inhibits the sphingosine kinase 1/sphingosine‐1‐phosphate signaling pathway in rabbits with fulminant hepatitis of viral origin. Journal of Pineal Research, 61, 168–176. 10.1111/jpi.12335 27101794

[jcp30735-bib-0011] Czechowska, G. , Celinski, K. , Korolczuk, A. , Wojcicka, G. , Dudka, J. , Bojarska, A. , & Reiter, R. J. (2015). Protective effects of melatonin against thioacetamide‐induced liver fibrosis in rats. Journal of Physiology and Pharmacology, 66(4), 567–579. 26348081

[jcp30735-bib-0012] Das, N. , Mandala, A. , Naaz, S. , Giri, S. , Jain, M. , Bandyopadhyay, D. , Reiter, R. J. , & Roy, S. S. (2017). Melatonin protects against lipid‐induced mitochondrial dysfunction in hepatocytes and inhibits stellate cell activation during hepatic fibrosis in mice. Journal of Pineal Research, 62(4), e12404. 10.1111/jpi.12404 28247434

[jcp30735-bib-0013] El‐Magd, M. A. , Mohamed, Y. , El‐Shetry, E. S. , Elsayed, S. A. , Abo Gazia, M. , Abdel‐Aleem, G. A. , Shafik, N. M. , Abdo, W. S. , El‐Desouki, N. I. , & Basyony, M. A. (2019). Melatonin maximizes the therapeutic potential of non‐preconditioned MSCs in a DEN‐induced rat model of HCC. Biomedicine and Pharmacotherapy, 114, 108732. 10.1016/j.biopha.2019.108732 30925457

[jcp30735-bib-0014] Esrefoglu, M. , Cetin, A. , Taslidere, E. , Elbe, H. , Ates, B. , Tok, O. E. , & Aydin, M. S. (2017). Therapeutic effects of melatonin and quercetin in improvement of hepatic steatosis in rats through supression of oxidative damage. Bratislava Medical Journal, 118(6), 347–354. 10.4149/BLL_2017_066 28664744

[jcp30735-bib-0015] González‐Fernández, B. , Sánchez, D. I. , Crespo, I. , San‐Miguel, B. , Álvarez, M. , Tuñón, M. J. , & González‐Gallego, J. (2017). Inhibition of the SphK1/S1P signaling pathway by melatonin in mice with liver fibrosis and human hepatic stellate cells. Biofactors, 43(2), 272–282. 10.1002/biof.1342 27801960

[jcp30735-bib-0016] González‐Fernández, B. , Sánchez, D. I. , Crespo, I. , San‐Miguel, B. , de Urbina, J. O. , González‐Gallego, J. , & Tuñón, M. J. (2018). Melatonin attenuates dysregulation of the circadian clock pathway in mice with CCl_4_‐induced fibrosis and human hepatic stellate cells. Frontiers in Pharmacology, 9, 556. 10.3389/fphar.2018.00556 29892224 PMC5985434

[jcp30735-bib-0017] Haeger, P. , Bouchet, A. , Ossandon, C. , & Bresky‐Ruiz, C. G. (2019). Treatment with melatonin improves cognitive behavior and motor skills in a rat model of liver fibrosis. Annals of Hepatology, 18(1), 101–108. 10.5604/01.3001.0012.7867 31113577

[jcp30735-bib-0018] Hu, W. , Ma, Z. , Jiang, S. , Fan, C. , Deng, C. , Yan, X. , Di, S. , Lv, J. , Reiter, R. J. , & Yang, Y. (2016). Melatonin: The dawning of a treatment for fibrosis? Journal of Pineal Research, 60(2), 121–131. 10.1111/jpi.12302 26680689

[jcp30735-bib-0019] Jiang, X. P. , Ai, W. B. , Wan, L. Y. , Zhang, Y. Q. , & Wu, J. F. (2017). The roles of microRNA families in hepatic fibrosis. Cell and Bioscience, 7, 34. 10.1186/s13578-017-0161-7 28680559 PMC5496266

[jcp30735-bib-0020] Joshi, A. , Upadhyay, K. K. , Vohra, A. , Shirsath, K. , & Devkar, R. (2021). Melatonin induces Nrf2‐HO‐1 reprogramming and corrections in hepatic core clock oscillations in non‐alcoholic fatty liver disease. FASEB Journal, 35, e21803. 10.1096/fj.202002556RRR 34365685

[jcp30735-bib-0021] Kang, J. W. , Cho, H. I. , & Lee, S. M. (2014). Melatonin inhibits mTOR‐dependent autophagy during liver ischemia/reperfusion. Cellular Physiology and Biochemistry, 33(1), 23–36. 10.1159/000356647 24401531

[jcp30735-bib-0022] Kang, J. W. , Hong, J. M. , & Lee, S. M. (2016). Melatonin enhances mitophagy and mitochondrial biogenesis in rats with carbon tetrachloride‐induced liver fibrosis. Journal of Pineal Research, 60(4), 383–393. 10.1111/jpi.12319 26882442

[jcp30735-bib-0023] Kennaway, D. J. (2020). Measuring melatonin by immunoassay. Journal of Pineal Research, 69, e12657. 10.1111/jpi.12657 32281677

[jcp30735-bib-0024] Laliena, A. , San‐Miguel, B. , Crespo, I. , Álvarez, M. , González‐Gallego, J. , & Tuñón, M. J. (2012). Melatonin attenuates inflammation and promotes regeneration in rabbits with fulminant hepatitis of viral origin. Journal of Pineal Research, 53(3), 270–278. 10.1111/j.1600-079X.2012.00995.x 22506987

[jcp30735-bib-0025] Lebda, M. A. , Sadek, K. M. , Abouzed, T. K. , Tohamy, H. G. , & El‐Sayed, Y. S. (2018). Melatonin mitigates thioacetamide‐induced hepatic fibrosis via antioxidant activity and modulation of proinflammatory cytokines and fibrogenic genes. Life Sciences, 192, 136–143. 10.1016/j.lfs.2017.11.036 29180002

[jcp30735-bib-0026] Lee, Y. A. , Wallace, M. C. , & Friedman, S. L. (2015). Pathobiology of liver fibrosis: A translational success story. Gut, 64(5), 830–841. 10.1136/gutjnl-2014-306842 25681399 PMC4477794

[jcp30735-bib-0027] Li, D. J. , Tong, J. , Li, Y. H. , Meng, H. B. , Ji, Q. X. , Zhang, G. Y. , Zhu, J. H. , Zhang, W. J. , Zeng, F. Y. , Huang, G. , Hua, X. , Shen, F. M. , & Wang, P. (2019). Melatonin safeguards against fatty liver by antagonizing TRAFs‐mediated ASK1 deubiquitination and stabilization in a β‐arrestin‐1 dependent manner. Journal of Pineal Research, 67(4), e12611. 10.1111/jpi.12611 31541591

[jcp30735-bib-0028] Li, H. W. , Ying, P. , Cai, Q. Q. , Yang, Z. H. , & Wu, X. L. (2021). Exogenous melatonin alleviates hemorrhagic shock‑induced hepatic ischemic injury in rats by inhibiting the NF‑κB/IκBα signaling pathway. Molecular Medicine Reports, 23(5), 341. 10.3892/mmr.2021.11980 33760198 PMC7974417

[jcp30735-bib-0029] Liao, N. , Shi, Y. , Wang, Y. , Liao, F. , Zhao, B. , Zheng, Y. , Zheng, Y. , Liu, X. , & Liu, J. (2020). Antioxidant preconditioning improves therapeutic outcomes of adipose tissue‐derived mesenchymal stem cells through enhancing intrahepatic engraftment efficiency in a mouse liver fibrosis model. Stem Cell Research and Therapy, 11(1), 237. 10.1186/s13287-020-01763-y 32546282 PMC7298967

[jcp30735-bib-0030] Malhotra, S. , Sawhney, G. , & Pandhi, P. (2004). The therapeutic potential of melatonin: A review of the science. Medscape General Medicine, 6(2), 46. PMC1395802 15266271

[jcp30735-bib-0031] Mallat, A. , Lodder, J. , Teixeira‐Clerc, F. , Moreau, R. , Codogno, P. , & Lotersztajn, S. (2014). Autophagy: A multifaceted partner in liver fibrosis. BioMed Research International, 2014, 869390. 10.1155/2014/869390 25254217 PMC4164803

[jcp30735-bib-0032] Mauriz, J. L. , Collado, P. S. , Veneroso, C. , Reiter, R. J. , & González‐Gallego, J. (2013). A review of the molecular aspects of melatonin's anti‐inflammatory actions: Recent insights and new perspectives. Journal of Pineal Research, 54(1), 1–14. 10.1111/j.1600-079X.2012.01014.x 22725668

[jcp30735-bib-0033] McMillin, M. , DeMorrow, S. , Glaser, S. , Venter, J. , Kyritsi, K. , Zhou, T. , Grant, S. , Giang, T. , Greene, J. F. , Wu, N. , Jefferson, B. , Meng, F. , & Alpini, G. (2017). Melatonin inhibits hypothalamic gonadotropin‐releasing hormone release and reduces biliary hyperplasia and fibrosis in cholestatic rats. American Journal of Physiology—Gastrointestinal and Liver Physiology, 313(5), G410–G418. 10.1152/ajpgi.00421.2016 28751425 PMC5792219

[jcp30735-bib-0034] Mortezaee, K. , & Khanlarkhani, N. (2018). Melatonin application in targeting oxidative‐induced liver injuries: A review. Journal of Cellular Physiology, 233(5), 4015–4032. 10.1002/jcp.26209 29023783

[jcp30735-bib-0035] Mortezaee, K. , Khanlarkhani, N. , Sabbaghziarani, F. , Nekoonam, S. , Majidpoor, J. , Hosseini, A. , Pasbakhsh, P. , Kashani, I. R. , & Zendedel, A. (2017). Preconditioning with melatonin improves therapeutic outcomes of bone marrow‐derived mesenchymal stem cells in targeting liver fibrosis induced by CCl_4_ . Cell and Tissue Research, 369(2), 303–312. 10.1007/s00441-017-2604-1 28413861

[jcp30735-bib-0036] Mortezaee, K. , Majidpoor, J. , Daneshi, E. , Abouzaripour, M. , & Abdi, M. (2018). Post‐treatment of melatonin with CCl_4_ better reduces fibrogenic and oxidative changes in liver than melatonin co‐treatment. Journal of Cellular Biochemistry, 119(2), 1716–1725. 10.1002/jcb.26331 28782839

[jcp30735-bib-0037] Mortezaee, K. , Pasbakhsh, P. , Kashani, I. R. , Sabbaghziarani, F. , Omidi, A. , Zendedel, A. , Ghasemi, S. , & Dehpour, A. R. (2016). Melatonin pretreatment enhances the homing of bone marrow‐derived mesenchymal stem cells following transplantation in a rat model of liver fibrosis. Iranian Biomedical Journal, 20(4), 207–216. 10.7508/ibj.2016.04.004 27130910 PMC4983675

[jcp30735-bib-0038] Nalobin, D. S. , Suprunenko, E. A. , & Golichenkov, V. A. (2016). Effects of melatonin on differentiation potential of Ito cells in mice with induced fibrosis of the liver. Bulletin of Experimental Biology and Medicine, 161(6), 845–849. 10.1007/s10517-016-3526-8 27783282

[jcp30735-bib-0039] Ostrycharz, E. , Wasik, U. , Kempinska‐Podhorodecka, A. , Banales, J. M. , Milkiewicz, P. , & Milkiewicz, M. (2020). Melatonin protects cholangiocytes from oxidative stress‐induced proapoptotic and proinflammatory stimuli via miR‐132 and miR‐34. International Journal of Molecular Sciences, 21(24), 9667. 10.3390/ijms21249667 PMC7766218 33352965

[jcp30735-bib-0040] Page, M. J. , McKenzie, J. E. , Bossuyt, P. M. , Boutron, I. , Hoffmann, T. C. , Mulrow, C. D. , Shamseer, L. , Tetzlaff, J. M. , Akl, E. A. , Brenna, S. E. , Chou, R. , Glanville, J. , Grimshaw, J. M. , Hróbjartsson, A. , Lalu, M. M. , Li, T. , Loder, E. W. , Mayo‐Wilson, E. , McDonald, S. , … Moher, D. (2021). The PRISMA 2020 statement: An updated guideline for reporting systematic reviews. Systematic Reviews, 10, 89. 10.1186/s13643-021-01626-4 33781348 PMC8008539

[jcp30735-bib-0041] Reiter, R. J. , Sharma, R. , Simko, F. , Dominguez‐Rodriguez, A. , Tesarik, J. , Neel, R. L. , Slominski, A. T. , Kleszczynski, K. , Martin‐Gimenez, V. M. , Manucha, W. , & Cardinali, D. P. (2022). Melatonin: Highlighting its use as a potential treatment for SARS‐CoV‐2 infection. Cellular and Molecular Life Sciences, 79, 143. 10.1007/s00018-021-04102-3 35187603 PMC8858600

[jcp30735-bib-0042] Renzi, A. , Glaser, S. , DeMorrow, S. , Mancinelli, R. , Meng, F. , Franchitto, A. , Venter, J. , White, M. , Francis, H. , Han, Y. , Alvaro, D. , Gaudio, E. , Carpino, G. , Ueno, Y. , Onori, P. , & Alpini, G. (2011). Melatonin inhibits cholangiocyte hyperplasia in cholestatic rats by interaction with MT1 but not MT2 melatonin receptors. American Journal of Physiology—Gastrointestinal and Liver Physiology, 301(4), 634–643. 10.1152/ajpgi.00206.2011 PMC3191552 21757639

[jcp30735-bib-0043] Reyes‐Gordillo, K. , Shah, R. , & Muriel, P. (2017). Oxidative stress and inflammation in hepatic diseases: Current and future therapy. Oxidative Medicine and Cellular Longevity, 2017, 3140673. 10.1155/2017/3140673 28203318 PMC5292177

[jcp30735-bib-0044] Roehlen, N. , Crouchet, E. , & Baumen, T. E. (2020). Liver fibrosis: Mechanistic concepts and therapeutic perspectives. Cells, 9, 875. 10.3390/cells9040875 PMC7226751 32260126

[jcp30735-bib-0045] San‐Miguel, B. , Crespo, I. , Sánchez, D. I. , González‐Fernández, B. , Ortiz De Urbina, J. J. , Tuñón, M. J. , & González‐Gallego, J. (2015). Melatonin inhibits autophagy and endoplasmic reticulum stress in mice with carbon tetrachloride‐induced fibrosis. Journal of Pineal Research, 59(2), 151–162. 10.1111/jpi.12247 25958928

[jcp30735-bib-0046] Sánchez, D. I. , González‐Fernández, B. , Crespo, I. , San‐Miguel, B. , Álvarez, M. , González‐Gallego, J. , & Tuñón, M. J. (2018). Melatonin modulates dysregulated circadian clocks in mice with diethylnitrosamine‐induced hepatocellular carcinoma. Journal of Pineal Research, 65(3), e12506. 10.1111/jpi.12506 29770483

[jcp30735-bib-0047] Shajari, S. , Laliena, A. , Heegsma, J. , Tuñón, M. J. , Moshage, H. , & Faber, K. N. (2015). Melatonin suppresses activation of hepatic stellate cells through RORα‐mediated inhibition of 5‐lipoxygenase. Journal of Pineal Research, 59(3), 391–404. 10.1111/jpi.12271 26308880

[jcp30735-bib-0048] Stacchiotti, A. , Grossi, I. , García‐Gómez, R. , Patel, G. A. , Salvi, A. , Lavazza, A. , Petro, G. De , Monsalve, M. , & Rezzani, R. (2019). Melatonin effects on non‐alcoholic fatty liver disease are related to microRNA‐34a‐5p/Sirt1 axis and autophagy. Cells, 8, 1053. 10.3390/cells8091053 PMC6770964 31500354

[jcp30735-bib-0049] Wang, Y. , Hong, R. , Xie, Y. , & Xu, J. (2018). Melatonin ameliorates liver fibrosis induced by carbon tetrachloride in rats via inhibiting TGF‐β1/Smad signaling pathway. Current Medical Science, 38(2), 236–244. 10.1007/s11596-018-1871-8 30074181

[jcp30735-bib-0050] Wu, N. , Meng, F. , Zhou, T. , Han, Y. , Kennedy, L. , Venter, J. , Francis, H. , DeMorrow, S. , Onori, P. , Invernizzi, P. , Bernuzzi, F. , Mancinelli, R. , Gaudio, E. , Franchitto, A. , Glaser, S. , & Alpini, G. (2017). Prolonged darkness reduces liver fibrosis in a mouse model of primary sclerosing cholangitis by miR‐200b down‐regulation. FASEB Journal, 31(10), 4305–4324. 10.1096/fj.201700097R 28634212 PMC5987749

[jcp30735-bib-0051] Yang, Z. , He, Y. , Wang, H. , & Zhang, Q. (2021). Protective effect of melatonin against chronic cadmium‐induced hepatotoxicity by suppressing oxidative stress, inflammation, and apoptosis in mice. Ecotoxicology and Environmental Safety, 228, 112947. 10.1016/j.ecoenv.2021.112947 34736034

[jcp30735-bib-0052] Zaitone, S. , Hassan, N. , El‐Orabi, N. , & El‐Awady, E. S. (2011). Pentoxifylline and melatonin in combination with pioglitazone ameliorate experimental non‐alcoholic fatty liver disease. European Journal of Pharmacology, 662, 70–77. 10.1016/j.ejphar.2011.04.049 21549113

[jcp30735-bib-0053] Zhang, S. , Chen, S. , Li, Y. , & Liu, Y. (2017). Melatonin as a promising agent of regulating stem cell biology and its application in disease therapy. Pharmacological Research, 117, 252–260. 10.1016/j.phrs.2016.12.035 28042087

